# Electrostatic Patterning of Nanofibrous Microcapsules for Three-Dimensional Cell Culture

**DOI:** 10.3390/jfb17010042

**Published:** 2026-01-15

**Authors:** Masashi Ikeuchi, Yoshinori Inoue, Ryosuke Tane, Daisuke Ishikawa, Chihiro Aoyama, Yoshitaka Miyamoto, Koji Ikuta

**Affiliations:** 1 Laboratory for Biomaterials and Bioengineering, Institute of Science Tokyo, Tokyo 113-8519, Japanyoshitaka.miyamoto@tmd.ac.jp (Y.M.); 2 School of Medical Science, Fujita Health University, Toyoake 470-1192, Japan; yoshinori.inoue@fujita-hu.ac.jp; 3 Graduate School of Information Science and Technology, The University of Tokyo, Tokyo 113-8657, Japanikuta@rcast.u-tokyo.ac.jp (K.I.)

**Keywords:** patterning, electrospray, electrospinning, nanofiber, scaffold

## Abstract

Three-dimensional biomaterial scaffolds with controlled geometry and surface nanoarchitecture are essential for advancing polymer processing strategies in tissue engineering. Conventional electrospinning generates nanofibrous structures but has limited ability to reproduce defined three-dimensional shapes or achieve high pattern fidelity. This study aimed to develop a scalable processing method for producing biodegradable scaffolds with precisely controlled microstructure and geometry using phase separation–assisted electrospray. Poly (lactic acid) microcapsules with tunable diameters and porous nanofibrous surfaces were fabricated under controlled humidity and deposited onto conductive molds to obtain two- and three-dimensional scaffold shapes. The manufacturing process required only simple electrospray equipment and static molds, without mechanically complex collectors or moving stages. The resulting scaffolds replicated mold features with resolutions down to 200 μm and achieved thickness up to 600 μm. The nanofibrous microcapsule surfaces supported strong adhesion and metabolic activity of HepG2 cells, while cellular penetration into deeper scaffold regions remained limited to approximately 80 μm. These findings indicate that electrospray-mediated microcapsule deposition is a practical polymer-processing approach that integrates nanofibrous surface formation with mold-defined shaping, offering a reproducible and scalable method for fabricating structurally precise and biologically compatible three-dimensional scaffolds.

## 1. Introduction

Tissue engineering has emerged as an interdisciplinary field that aims to restore or replace damaged tissues by combining cells, biomaterials, and bioactive factors within a three-dimensional (3D) microenvironment [1,2,3,4]. In native tissues, the scaffold is the extracellular matrix (ECM). Structural ECM proteins are recognized by cells as thin fibers of 50–500 nm, 1 to 2 orders of magnitude smaller than the cell itself. This allows the cell to be in direct contact with many ECM fibers, thus permitting cell/cell or cell/scaffold interactions [5]. Nanoscale artificial architectures make the cell create more filopodia and increase the proliferation and attachment ability of the cell [6,7,8]. Various techniques have been developed to fabricate scaffolds that mimic the structural and functional features of the native ECM, including self-assembly [9,10], phase separation [11,12], and electrospinning [13,14]. Among these, electrospinning has become one of the most widely used approaches because it enables the fabrication of micro- to nanoscale fibrous networks with high surface area, interconnected porosity, and tunable mechanical properties, closely resembling the fibrous architecture of native ECM. Recent reviews have highlighted how electrospun scaffolds have been applied across diverse biomedical fields such as tissue engineering, wound healing, drug delivery, and biosensing, and have emphasized their advantages in terms of structural design and functionalization [15,16,17,18]. In particular, electrospun nanofiber scaffolds have been increasingly exploited as 3D culture platforms for epithelial cells, liver-derived cells, and cancer cells, where they support cell adhesion, proliferation, and maintenance of tissue-specific functions under physiologically relevant conditions [19,20,21]. These developments suggest that electrospun fibrous matrices provide a promising basis for constructing in vitro models that better recapitulate tissue microenvironments compared with conventional two-dimensional (2D) culture substrates.

Despite these advantages, conventional electrospinning still faces inherent limitations when used to fabricate three-dimensional (3D) culture platforms. Electrospun nanofibers are continuously connected and highly entangled, which restricts structural control and prevents the formation of well-defined microarchitectures in the vertical direction. As scaffold thickness increases, pore collapse frequently occurs between the layers, resulting in limited inter-fiber spacing and poor medium permeability, thereby inhibiting sufficient cell infiltration and oxygen–nutrient transport [22,23,24]. Beyond mean pore size, 3D microstructural analyses have shown that pore shape and local connectivity critically govern cell ingrowth in electrospun fiber networks, helping explain why thick electrospun constructs often exhibit superficial colonization [25]. Recent efforts to generate 3D electrospun constructs—such as multilayer stacking, patterned collectors, or post-processing expansion—have shown partial improvements but still suffer from non-uniform porosity, low fabrication speed, and restricted geometric precision, particularly when constructing complex microscale contours [17,19,26,27]. Even advanced approaches integrating decellularized ECM or aligned nanofibers to promote hepatocyte and epithelial cell functions have been unable to overcome the bottleneck of producing thick, shape-specific scaffolds with open, vertically continuous pores [20,21,28]. Therefore, there remains a critical need for a fabrication method capable of generating biodegradable 3D scaffolds with controlled microscale topography, reliable vertical porosity, and applicability to cell culture systems requiring efficient nutrient diffusion and structural fidelity.

To address these limitations, the present study introduces a scalable fabrication strategy that exploits phase separation–assisted electrospray to generate biodegradable nanofibrous microcapsules and directly deposit them onto reusable conductive molds. Unlike continuous electrospun fibers, the microcapsules behave as discrete, porous building blocks that maintain vertically open pathways and enable precise microscale patterning in both two- and three-dimensional configurations [29]. Therefore, this approach overcomes the geometric constraints inherent to conventional electrospinning and allows the rapid formation of thick, shape-specific scaffolds with interconnected nanofibrous surfaces suitable for cell adhesion. Using poly(lactic acid) as a model material, we demonstrate the fabrication of microstructured scaffolds and evaluate their cytocompatibility through human liver cancer cell line HepG2 cell culture and proliferation assays. Collectively, this study establishes a mass-producible platform for constructing 3D nanofibrous scaffolds with high architectural fidelity, thereby offering an alternative route for developing physiologically relevant culture systems and advanced tissue-engineering constructs.

## 2. Materials and Methods

### 2.1. Materials for Nanofibrous Microcapsule

Poly(lactic acid) (PLA; inherent viscosity 4.52 dL/g) was obtained from Corbion Purac (Amsterdam, The Netherlands) and used as the primary polymer for scaffold fabrication. Chloroform (analytical grade) and ethanol (99.5%) were purchased from Fujifilm-Wako Pure Chemical Corporation (Tokyo, Japan). Polyvinyl alcohol (PVA; molecular weight 22,000–31,000, 87–89% hydrolyzed) was also obtained from Fujifilm-Wako and used as a water-soluble sacrificial layer for mold surface treatment. All other reagents were of analytical grade and used without further purification.

### 2.2. Preparation of Polymer Solution

According to our previous paper [29], the polymer solution for phase separation–assisted electrospray was prepared by dissolving PLA in chloroform under gentle agitation overnight at room temperature. After complete dissolution, ethanol was added to induce a controlled phase separation during droplet formation. The final composition of the solution was 1.0 wt% PLA, 94.0 wt% chloroform, and 5.0 wt% ethanol. The mixture was homogenized thoroughly before use and stored in a sealed glass vial to prevent solvent evaporation. As reference experiments, another solution for electrospinning nanofibers was prepared consisting of 2.0 wt% PLA, 78.0 wt% chloroform and 20.0 wt% ethanol.

### 2.3. Design and Fabrication of Conductive Molds

Conductive molds used for two-dimensional and three-dimensional scaffold fabrication were produced using either aluminum or photocurable resin depending on the target geometry. For two-dimensional scaffolds, molds were machined from 1-mm-thick aluminum plates using wire electrical discharge machining to create defined microstructured patterns. For three-dimensional scaffolds, molds were fabricated from a photocurable resin (SCR770, D-MEC, Tokyo, Japan) via microstereolithography. The resin molds were subsequently coated with a thin layer of gold using an ion sputtering system to ensure uniform conductivity across the mold surface (Supplementary Figure S1). Prior to electrospray deposition, each mold was dip-coated in an aqueous polyvinyl alcohol (PVA) solution and air-dried to form a sacrificial, water-soluble release layer. This treatment enabled clean detachment of the fragile nanofibrous microcapsule scaffolds after fabrication. Polyvinyl alcohol (PVA) was used as the sacrificial layer because of its cytocompatibility and high solubility in water.

### 2.4. Electrostatic Scaffold Fabrication Process

**Figure 1:**
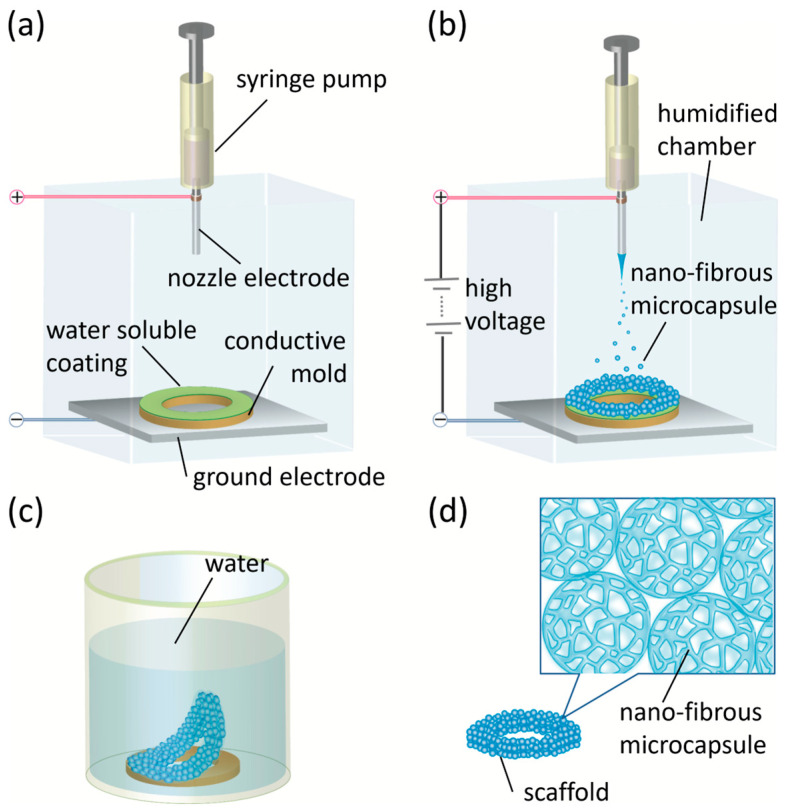
3-D scaffold fabrication using phase separation-assisted electrospray. (**a**) A syringe pump with a steel nozzle was placed in a humidified chamber and connected to a positive electrode. A conductive mold coated with PVA was fixed on a flat ground electrode perpendicular to the nozzle. (**b**) The syringe was filled with polymer solution and high voltage was applied between the nozzle and the ground electrode. Since the mold was conductive, the electric field was higher on the mold, making the nanofibrous microcapsules accumulate on the surface of the mold. (**c**) After electrospraying, the molds were sunk in ultrapure water, defoamed in a vacuum chamber, and left overnight to allow the sacrificial PVA layer to dissolve and cause the scaffold to detach from the mold naturally. (**d**) The scaffold composed of nanofibrous microcapsules was completed by drying in a vacuum.

Scaffolds were fabricated by depositing nanofibrous microcapsules directly onto the conductive molds using a custom-built electrospray apparatus configured inside a humidity-controlled chamber for stable electrospray operation (Figure 1) [29]. A stainless-steel nozzle was connected to a high-voltage power supply and operated at +20 kV. The conductive mold was fixed onto a grounded metal plate positioned perpendicular to the electrospray nozzle. The polymer solution was delivered through the nozzle at a constant flow rate of 2.4 mL/h using a syringe pump. The working distance between the nozzle tip and the grounded conductive plate was maintained at 180 mm. The chamber temperature and relative humidity were controlled at 22 °C and 80% RH, respectively, to promote vapor-induced phase separation during droplet flight. Under these conditions, the positively charged polymer jet fragmented into microdroplets due to electrostatic repulsion. Water vapor condensed on the droplet surface caused phase separation between the organic-rich PLA phase and the aqueous phase, leading to the formation of a nanofibrous polymer network. After complete evaporation of the solvents, the microdroplets solidified into porous nanofibrous microcapsules, which were collected directly on the conductive mold surface during scaffold fabrication.

Upon completing the electrospray deposition, the mold containing the newly formed scaffold was immersed in ultrapure water and placed inside a vacuum chamber to remove trapped air and accelerate dissolution of the PVA sacrificial layer. The scaffold detached smoothly from the mold after overnight immersion with just gentle pipetting. The released scaffold was then collected with a flat tip tweezers and dried under vacuum to remove residual moisture before further characterization and cell culture experiments. Scaffold thickness was controlled by adjusting the electrospray duration.

### 2.5. Microscopy and Imaging

Structural characterization of the nanofibrous microcapsule scaffolds was performed using both scanning electron microscopy (SEM) and digital optical microscopy. SEM imaging was conducted using scanning electron microscopes (JSM-7500F or JSM-IT200LA, JEOL, Tokyo, Japan). Samples were sputter-coated with a thin gold layer prior to observation, and images were acquired at accelerating voltages suitable for polymeric materials to visualize microcapsule morphology, scaffold cross-sections, and cell–scaffold interfaces. Complementary surface and structural evaluations were performed using a digital microscope (VHX-2000, Keyence, Osaka, Japan) under high-resolution optical imaging modes.

### 2.6. Tensile Test

To investigate the tensile strength of the nanofibrous microcapsule scaffold, scaffolds with a width of 3 mm, a length of 10 mm, and a thickness of 0.2 mm were prepared (*n* = 3). One end of the scaffold was fixed to a jig directly connected to the force sensor (FS1M-0.1NBL, THK Precision, Tokyo, Japan), and the other end was fixed to the jig on the micro-motion automatic stage (OSMS 20-35, OptoSigma, Saitama, Japan). The scaffold was pulled at a speed of 10 µm/s, and the force acting on the scaffold during that time was recorded with a sampling period of 10 ms by using the amplifier (FSA201CL, THK Precision, Tokyo, Japan).

### 2.7. Cell Culture

Prior to cell seeding, the fabricated scaffolds were coated with type I bovine collagen to promote cell adhesion. Collagen (Cellmatrix Type I, Nitta-Gelatin, Osaka, Japan) was diluted to a concentration of 50 μg/mL in 0.01 M HCl, applied to the scaffold surface, and incubated for 1 h at room temperature. Excess collagen solution was removed, and the scaffolds were rinsed twice with phosphate-buffered saline (PBS). HepG2 cells (purchased from the American Type Culture Collection, ATCC, Manassas, VA, USA) were cultured under standard conditions in a humidified incubator at 37 °C with 5% CO_2_. Cells were seeded onto each scaffold placed in a 35 mm culture dish at a density of 8.3 × 10^4^ cells/mm^2^. The culture medium was replaced once after 24 h, and cells were maintained for 96 h before analysis.

### 2.8. Cell Viability and Proliferation Assays

Cell viability on the nanofibrous microcapsule scaffolds was assessed after 96 h of culture using a standard MTT assay (Cell Proliferation Kit 1 (MTT), Roche Applied Science, Basel, Switzerland). Following the removal of the culture medium, 100 μL of MTT solution (0.5 mg/mL in culture medium) was added to each scaffold-containing dish and incubated for 4 h at 37 °C. Metabolically active cells reduced MTT to insoluble formazan crystals, which were subsequently dissolved in dimethyl sulfoxide. The optical density (OD) of the resulting solution was measured at 570 nm with background subtraction at 690 nm using a spectrophotometer (UV-1900, Shimadzu, Kyoto, Japan). All measurements were performed in triplicate. Collagen-coated standard tissue culture plates were used as controls under identical assay conditions. Quantitative data from the MTT assays were presented as mean ± standard deviation from three independent samples (*n* = 3). All measurements were evaluated under identical experimental conditions. A significance threshold was not applied because the objective of the assay was to compare relative metabolic activity rather than to conduct hypothesis testing.

### 2.9. Live Cell Imaging

For visualization of cell viability, live/dead staining was performed following the 96 h culture period using Viability/Cytotoxicity Assay Kit (No.30002, Biotium, Fremont, CA, USA) consisting of Calcein-AM (live cells, green) and ethidium homodimer-1 (dead cells, red) according to the manufacturer’s protocol. Live-cell imaging was carried out using confocal laser scanning microscopy (FV-1200, Olympus, Tokyo, Japan) following Calcein-AM staining, enabling three-dimensional visualization of viable cells and assessment of their spatial distribution within the scaffold. Additionally, fluorescence images of live/dead-labeled cells were obtained using a digital fluorescence microscope (BZ-X700, Keyence, Osaka, Japan) to provide complementary wide-field imaging for qualitative evaluation. All fluorescence images were acquired under consistent exposure settings to ensure comparability across samples.

### 2.10. Histological Sectioning

To evaluate the depth of cellular infiltration into the nanofibrous microcapsule scaffolds, samples were fixed and embedded using a hydrogel-based embedding reagent (iPGell, Genostaff, Tokyo, Japan) to avoid the fracture of the micro-nano structures of the scaffold [30,31]. Following 96 h of cell culture, scaffolds were gently rinsed with PBS and fixed in 4% paraformaldehyde for 30 min at room temperature. Fixed samples were then immersed in iPGell according to the manufacturer’s protocol and allowed to polymerize to maintain the internal microarchitecture of the scaffold during sectioning. The embedded scaffolds were sectioned into 10-μm-thick slices. Sectioned samples were mounted on glass slides and subjected to standard hematoxylin and eosin (HE) staining. After dehydration and mounting, the stained sections were imaged using a digital microscope to evaluate the spatial distribution of cells within the scaffold. Cellular infiltration depth was quantified as the maximum distance from the scaffold surface at which cell nuclei were detectable in cross-sectional images.

## 3. Results

### 3.1. Two-Dimensional Scaffold Fabrication

**Figure 2:**
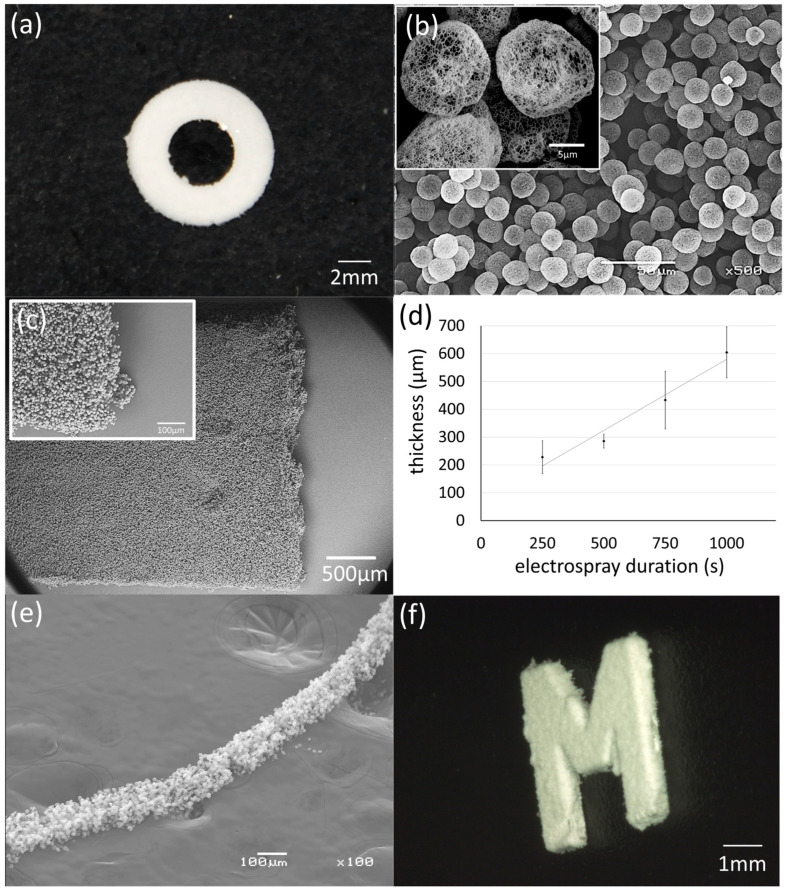
Scaffold fabrication with 2-D conductive molds using phase separation-assisted electrospray. (**a**) A scaffold fabricated using a ring-shaped conductive mold. (**b**) SEM observation showed that the entire scaffold was composed of uniform nanofibrous microcapsules. (**c**) SEM image of the test specimen after tensile test. Overall view of the fracture surface and a magnified image showing individual microcapsules. (**d**) Relationship between the electrospray duration and the thickness of the scaffold. The deposition speed was approximately 0.6 μm/s. (**e**) A thread scaffold composed of nanofibrous microcapsules fabricated using a φ100 μm metal wire as a conductive mold. (**f**) A 4 mm square “M” shaped scaffold of 600 μm thickness fabricated using a metal mold.

To evaluate pattern fidelity, a ring-shaped conductive mold (inner diameter 4 mm, outer diameter 8 mm) was employed. The resulting scaffold reproduced the mold geometry with high accuracy, forming a circular structure with an average thickness of approximately 170 μm on its flat regions (Figure 2a). Localized irregularities were observed near mold edges, where microcapsule bridging between the mold and the ground electrode produced small aggregated clusters. These edge effects likely arose from partial adhesion of microcapsules during deposition and was minimized by removing loosely attached particles through a stream of water during the scaffold detachment process in water. After the detachment process of the scaffold from the mold in water, no PVA residual on the scaffold surface was detected (Supplementary Figure S2).

SEM observations confirmed uniform packing of nanofibrous microcapsules across the scaffold surface (Figure 2b). The mesh structure composed of nanofibers with diameters ranging from 100 nm to 200 nm on the capsule exterior demonstrates that vapor-induced phase separation occurred successfully during droplet solidification. This observation confirms that the electrospray parameters were optimal for generating consistent nanofibrous microcapsule architectures (see Figure 2b inset, Supplementary Figure S3). The diameter of the microcapsules was controlled by the flow rate of the polymer solution supplied to the electrospray nozzle. At the flow rate of 2.4 mL/h, the average diameter of the microcapsules was 14.1 ± 2.3 μm, as quantified from SEM micrographs (Supplementary Table S1). The tensile strength of the achieved nanofibrous microcapsule scaffold was 34.8 ± 0.9 kPa with maximum elongation 37 ± 2%. At the final fracture surface, little plastic deformation or failure of the microcapsules themselves was observed; instead, failure appeared to occur at the adhesive interface between the microcapsules. (Figure 2c). The thickness of the nanofibrous microcapsule scaffolds increased proportionally with electrospray duration, enabling precise thickness control (Figure 2d). Quantitative analysis revealed a deposition rate of approximately 0.6 μm/s, allowing the fabrication of scaffolds up to 600 μm thick within 1000 s. For further validation of the process, a 100 μm wide strip-like structure composed of the nanofibrous microcapsules was fabricated using a ϕ100 μm metal wire as a mold (Figure 2e). Also, a “M” shaped scaffold was fabricated and accurately reflected the mold shape (Figure 2f).

### 3.2. Three-Dimensional Scaffold Fabrication and Patterning

**Figure 3:**
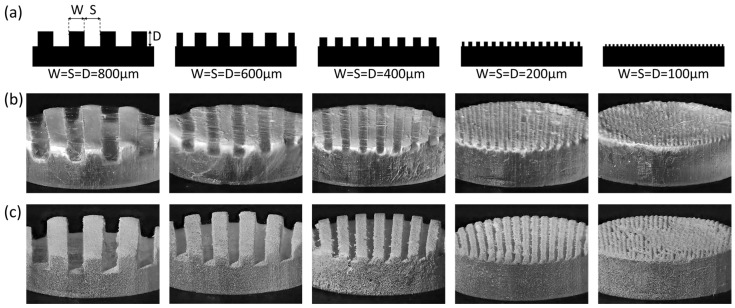
Comparison of gap-filling ability between the nanofibers and nanofibrous microcapsules. (**a**) Design of the conductive molds (**b**) Microscopy images of the molds after nanofibers were deposited by electrospinning. Those molds are arranged in the same order as shown in (**a**). The nanofibers bridged over gaps and did not deposit on the bottom surface even with the largest mold with 800 μm width. (**c**) Microscopy images of the molds after nanofibrous microcapsules were deposited by electrospray. Nanofibrous microcapsules deposited in the gaps with the mold with 200 μm width.

The capability of the electrospray-based process to fabricate complex three-dimensional (3D) microstructured scaffolds was assessed using conductive molds with walls lined at 100, 200, 400, 600, and 800 μm intervals (Figure 3a). Nanofibers formed by conventional electrospinning covered the walls of every mold and could not fill the gaps between the walls lined at 800 μm intervals (Figure 3b). In contrast, nanofibrous microcapsules filled into the gaps and deposited according to the surface of the same mold (Figure 3c). However, in molds with narrow intervals (100, 200 μm), partial bridging occurred at the top edges, leading to non-uniform fillings near the uppermost region. This behavior suggests that microcapsule size and droplet trajectory impose practical resolution limits, with a minimum reproducible interval of approximately 200 μm under the present electrospray conditions.

**Figure 4:**
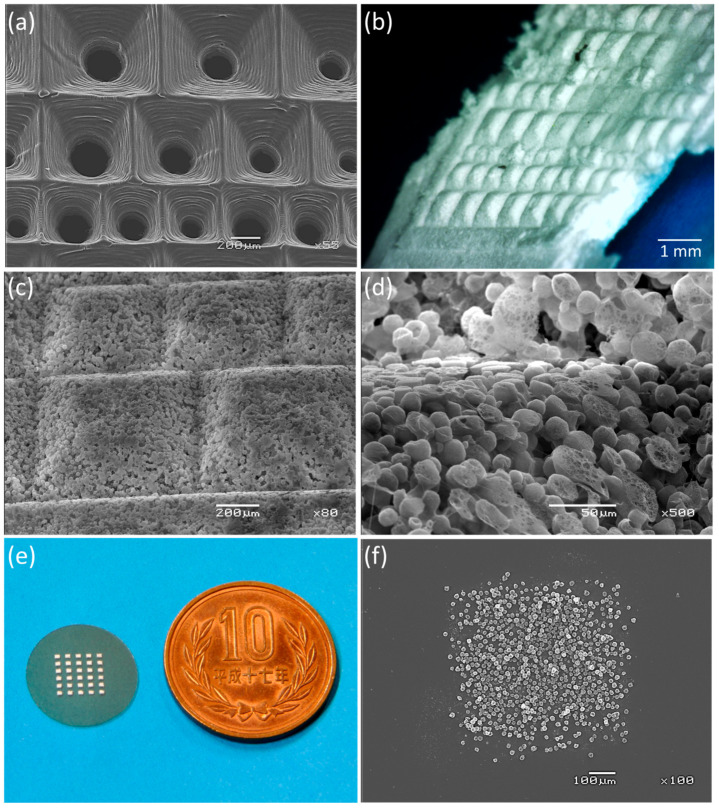
Three-dimensional scaffold fabrication and patterning with nanofibrous microcapsules. (**a**) SEM image showing an array of conductive molds with inverted pyramids in three sizes: square bases of 800 μm, 600 μm, and 400 μm per side, each with a depth of 500 μm. (**b**) Optical microscopy image of the scaffold after detaching from the mold. Note that the edges of the scaffold fell off during manipulation. (**c**) SEM image of the pyramidal structure composed of nanofibrous microcapsules. (**d**) Magnified SEM image of the corner of the pyramidal structure. Microcapsules are uniformly and densely deposited, extending fully into the corners of the mold. (**e**) A cover glass with the nanofibrous microcapsules patterned using a mask featuring 5 × 5 grid-arranged square through-holes measuring 600 μm per side. (**f**) Magnified SEM image of a deposited area on the cover glass. Patterning approximately reflects the mask area of 600 μm square.

To further evaluate the fabrication of complex geometries, three sizes of inverted pyramid-shaped conductive molds were prepared, each with a square base measuring 800 μm, 600 μm, and 400 μm per side, all with the same depth of 500 μm (Figure 4a). The 1000 s electrospraying process precisely reproduced the pyramid structure, forming a complete 3D scaffold composed solely of nanofiber-like microcapsules atop a common 100 μm-thick square base structure (Figure 4b). SEM analyses revealed a highly porous surface with interconnected micropores, indicating that the scaffold architecture was favorable for cell infiltration and nutrient diffusion (Figure 4c,d).

Another interesting application of this method is the patterning of the nanofibrous microcapsules onto another substrate. An electrically conductive mold with 600 μm square holes arranged in a 5 × 5 grid pattern was placed on an electrically conductive glass slide, and an electrospray was performed. As a result, the microcapsules filled the interior of the through holes. Upon removing the mold, a grid-like area precisely patterned with microcapsule deposits was formed on the slide glass (Figure 4e). Magnified SEM images reveal that while microcapsule distribution is non-uniform near the mask boundary, patterning accurately reflects the mask area of approximately 600 μm square (Figure 4f).

### 3.3. Cell Adhesion and Viability on the Scaffolds

**Figure 5:**
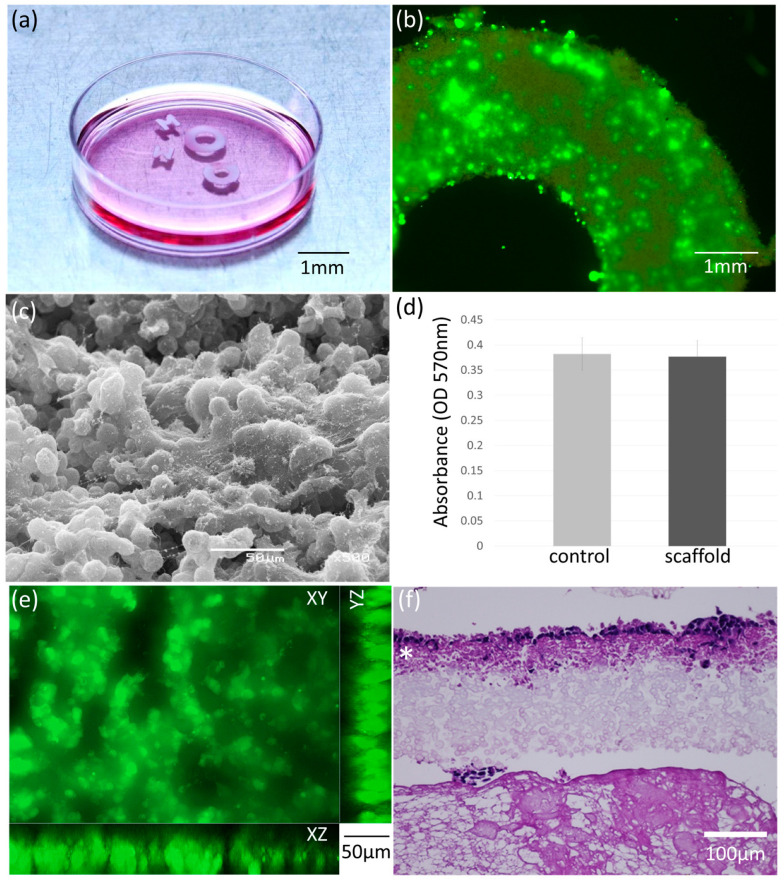
(**a**) Patterned scaffolds composed of nanofibrous microcapsules used for cell culture. (**b**) A Calcein-AM viability assay showing living cells on the scaffold. The adhered cells were shown to be viable and well spread on the scaffold surface. (**c**) SEM image of cells on the top surface of the scaffold (**d**) A MTT assay showed that the cell activity on the developed scaffold was approximately equal to that in normal cell culture dishes. (**e**) Reconstructed three-dimensional confocal microscopy image of living cells on the scaffold. Cells seemed to be present only within 100 μm below the top surface of the scaffold. (**f**) HE-stained section of the scaffold after culture. Cells were observed in the region extending approximately 80 μm deep from the surface (*), and in deeper regions, only unstained microcapsules were observed.

The cytocompatibility of the nanofibrous microcapsule scaffolds was evaluated through HepG2 cell adhesion and viability assays (Figure 5a). After 96 h of culture, live/dead staining revealed a high proportion of viable cells distributed across the scaffold surface (Figure 5b). Scanning electron microscopy clearly showed that cells adhered firmly to the nanofibrous exterior of the microcapsules and displayed a well-spread morphology (Figure 5c).

Quantitative assessment using the MTT assay further demonstrated that the metabolic activity of cells cultured on the scaffolds was comparable to that of cells grown on collagen-coated culture plates (Figure 5d). The spatial distribution of HepG2 cells within the nanofibrous microcapsule scaffolds was examined using confocal laser scanning microscopy. Three-dimensional confocal reconstructions revealed that viable cells were predominantly located within approximately 50–100 μm beneath the scaffold surface (Figure 5e). Only a small number of cells penetrated deeper regions of the scaffold, despite the presence of interconnected micropores within the microcapsule assemblies. Histological sectioning observations supported these findings, showing that cells strongly adhered to the outer surface of the microcapsules and formed intimate contacts with the nanofibrous network (Figure 5f). However, the deeper regions of the scaffold below 80 μm contained few attached cells.

## 4. Discussion

The microcapsules derived from phase separation assisted electrospray exhibited uniform geometry and ECM-like nanofibrous surfaces, while conductive molds allowed accurate replication of two- and three-dimensional features down to 200 μm. Compared to the direct 3-D printing of microcapsules [29], the simplicity of the electrospray setup, which requires no moving collectors or complex mechanical systems, further highlights its suitability for scalable scaffold production. Also, the rapid deposition rate of approximately 0.6 μm/s allowing the fabrication of thick scaffolds within a substantially shorter processing time than conventional electrospinning [32]. In contrast to various 3D electrospun thickening methods—which frequently necessitate supplementary post-processing steps and may affect shape-specific fidelity—it is notable that the use of microcapsules enables the simultaneous creation of both macroscale and nanoscale structures [26,27,33,34].

The high pattern fidelity is a direct consequence of the particle-like behavior of the microcapsules during deposition. In contrast to continuous electrospun fibers, which can suffer from jet instability, entanglement, and bridging over mold features, microcapsules move along electric-field gradients and settle into recessed areas of the mold. This allows for precise shaping, even with complex 3D structures. The resolution is mainly constrained by plume broadening and field disturbances at the edges—factors that become more significant as feature sizes near the spray footprint’s dimensions. Recent research indicates that edge non-uniformity can often be reduced through charge management and collector design approaches, such as focusing elements, grounding techniques, or insulator-guided deposition. These strategies offer practical solutions for enhancing boundary definition in future developments [35,36].

The tensile strength of the microcapsule scaffold was 1/10 to 1/100th of the tensile strength of electrospun PLA nanofiber scaffolds [37,38,39]. This is thought to be due to the fact that the void ratio in the thickness direction is larger than that of nanofiber scaffolds, and that the adhesion between microcapsules is weak and the adhesive surface area is small. To improve this strength issue, a possible future development would be to immerse the scaffold in a polymer solution such as collagen after creation to form a composite structure.

Hepatocyte culture results showed favorable spreading behavior, which is attributed to the nanoscale fibrous topography of the microcapsule surface since bulk PLA does not have ideal chemical properties for cell adhesion [40,41,42]. The high surface-to-volume ratio and interconnected nanofiber network may provide abundant anchoring sites for focal adhesion formation, promoting cytoskeletal organization and cell spreading [43]. Furthermore, the microscale curvature of the capsule surface may mitigate excessive cellular confinement, thereby facilitating stable cell–material interactions across multiple nanofibers. Confirming the exact mechanisms underlying cell–scaffold interactions necessitates single-cell level observations; however, this remains challenging due to the pronounced light scattering characteristics inherent to the nanofibrous microcapsule. Advancements in observational methodologies, including the application of tissue clearing techniques and/or two-photon excitation laser confocal microscopy, represent crucial next steps.

Despite the advances in creating intricate three-dimensional scaffolds for tissue culture, cell infiltration remains insufficient, likely due to restricted perfusion and inadequate oxygen delivery. Accordingly, integrating perfusable microchannels and/or dynamic culture systems represents a practical next step to mitigate transport limitations and enhance infiltration. Extending this approach to additional cell types, including primary or iPSC-derived hepatocytes, could broaden its applicability.

## 5. Conclusions

This study demonstrates that phase separation–assisted electrospray enables the fabrication of biodegradable three-dimensional scaffolds composed of nanofibrous microcapsules with microscale pattern fidelity that exceeds the capabilities of conventional electrospinning. The process generated microcapsules with controlled diameter and porous nanofibrous surfaces, which collectively provided an ECM-mimetic interface that supported robust HepG2 adhesion and proliferation. By depositing these microcapsules onto conductive molds, two- and three-dimensional scaffold geometries were reproduced with high accuracy, achieving resolutions down to 200 μm and scaffold thicknesses up to 600 μm. Overall, the findings establish electrospray-mediated microcapsule deposition as a scalable and mold-compatible strategy for constructing architecturally precise, ECM-like 3D scaffolds. Although applying this method to other biodegradable and bioderived polymers is still a challenge, it offers a practical alternative to conventional electrospinning and marks important progress toward reproducible, application-ready scaffolds for advanced cell culture and tissue engineering.

## Data Availability

The original contributions presented in this study are included in the article/Supplementary Materials. Further inquiries can be directed to the corresponding author.

## Supplementary Materials

The following supporting information can be downloaded at: https://www.mdpi.com/article/10.3390/jfb17010042/s1, Figure S1: Schematic of the fabrication process of three-dimensional conductive mold. Figure S2: FTIR spectra of scaffold with different rinse durations. The “*” symbol marks the peak corresponding to O–H stretching caused by hydrogen bonding. Figure S3: AFM images of a nanofibrous microcapsule in the scaffold. (a) Surface image of one microcapsule that makes up the scaffold. (b) Enlarged view of the area surrounded by white dashed lines in the image (a). Scale bar shows 2 μm and 1 μm, respectively. Table S1: The relationship between the flow rate of solution supplied to the electrospray nozzle and the resulting microcapsule diameter.
