# Supplementary material for: Electrostatic Patterning of Nanofibrous Microcapsules for Three-Dimensional Cell Culture
Source: J Funct Biomater. 2026 Jan 15;17(1):42. doi: 10.3390/jfb17010042 (PMC12842361; doi:10.3390/jfb17010042)
Supplement: Supplementary file 1 [file jfb-17-00042-s001.zip › jfb-4042185-supplementary.pdf]

Supplementary Materials

Electrostatic Patterning of Nanofibrous Microcapsules for Three-Dimensional Cell Culture

Masashi Ikeuchi [1,*], Yoshinori Inoue [2], Ryosuke Tane [3], Daisuke Ishikawa [1], Chihiro Aoyama [1], Yoshitaka Miyamoto [1] and Koji Ikuta [3]

[1] Laboratory for Biomaterials and Bioengineering, Institute of Science Tokyo, Tokyo 113-8519, Japan;
[2] School of Medical Science, Fujita Health University, Toyoake 470-1192, Japan;
[3] Graduate School of Information Science and Technology, The University of Tokyo, Tokyo 113-8657, Japan;
* Correspondence: ikeuchi@md.isct.ac.jp

1. Fabrication Process of the three-dimensional conductive mold

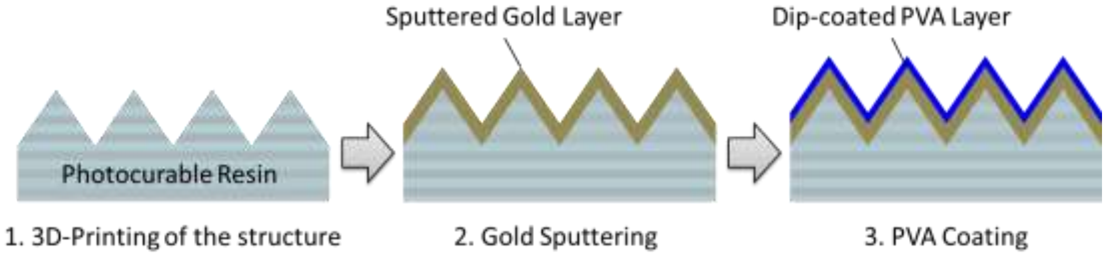

Figure S1. Schematic of the fabrication process of three-dimensional conductive mold

2. PVA residue testing by Fourier Transform Infrared (FTIR) spectroscopy

FTIR analysis was conducted with FT/IR-4700 (JASCO, Japan) to assess whether polyvinyl alcohol (PVA) from the mold coating persisted on the scaffolds after removal. Nanofibrous microcapsules were electrosprayed to an aluminum substrate at a thickness of 200 μm, creating sheet-like scaffolds. These scaffolds were then dip-coated in an aqueous PVA solution and left to air dry. This coating procedure could result in a higher amount of residual PVA within the scaffold compared to the method described in this paper. Samples included unrinsed scaffolds, those rinsed for 10 or 30 seconds, PVA film, and microcapsules without a PVA coating.

Supplementary Figure 2. Shows the FTIR spectra of the samples. The results showed a prominent peak around 3300 cm−1 in the PVA film (orange) and unrinsed scaffold (light blue), indicating O–H stretching due to hydrogen bonds (Supplementary Figure 2) (Herman S., 2008). This peak disappeared in samples washed for 10 seconds (light green) or 30 seconds (brown), whose spectra matched those of microcapsules without PVA (dark green). In the cell culture experiments, scaffolds were immersed in water for over 12 hours, making residual PVA negligible.

- Herman S. Mansur, Carolina M. Sadahira, Adriana N. Souza, Alexandra A.P. Mansur, FTIR spectroscopy characterization of poly (vinyl alcohol) hydrogel with different hydrolysis degree and chemically crosslinked with glutaraldehyde, *Materials Science and Engineering: C* 28(4), **2008**, 539-548, https://doi.org/10.1016/j.msec.2007.10.088.

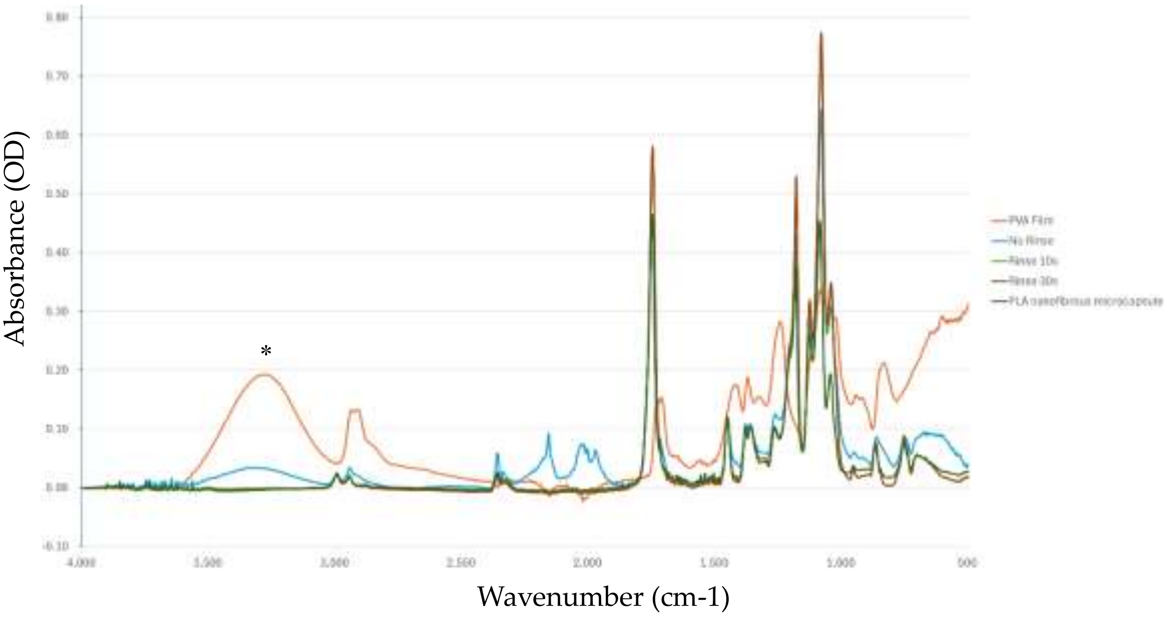

Figure S2. FTIR spectra of scaffold with different rinse durations. The “*” symbol marks the peak corresponding to O–H stretching caused by hydrogen bonding.

3. Atomic Force Microscopy (AFM) of the Electrosprayed Microcapsules

In SEM observation, it is difficult to observe the nanofiber structure magnified because the sample is easily deformed by the influence of heat. Therefore, in order to evaluate the nanofiber structure in a more natural state, AFM images were obtained using a NanoScope V instrument (Veeco Instruments Inc., USA) with a silicon cantilever (NCH Pointprobe, NanoWorld, Switzerland) in the tapping mode in air to characterize the surface topography of the samples. The tip curvature radius of the cantilever was <8 nm (<12 nm guaranteed). Supplementary Figure 3 shows a magnified view of the surface of the scaffold, which is composed of densely packed microcapsules. It is clear that the spherical shell of the microcapsule is formed by nanofibers with a diameter of 100 nm ~ 200 nm.

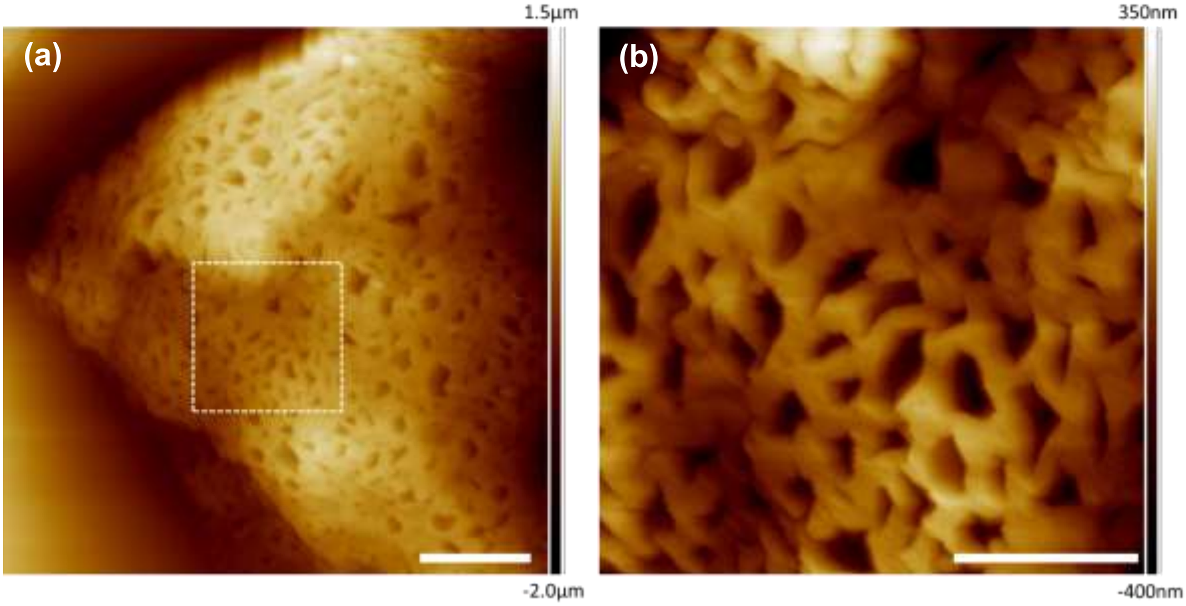

Figure S3. AFM images of a nanofibrous microcapsule in the scaffold. (a) Surface iamge of one microcapsule that makes up the scaffold. (b) Enlarged view of the area surrounded by white dashed lines in the image (a). Scale bar shows 2μm and 1μm, respectively.

Table S1. The relationship between the flow rate of solution supplied to the electrospray nozzle and the resulting microcapsule diameter.

Flow Rate (ml/h)	2.4	3.6	4.8	6.0	7.2
Microcapsule Diameter (μm)	14.1±2.3	16.7±2.5	17.1±1.7	18.0±1.7	19.6±1.5
